# Trauma induces apoptosis in human thoracolumbar intervertebral discs

**DOI:** 10.1186/1472-6890-6-5

**Published:** 2006-05-23

**Authors:** Christoph-E Heyde, Sven K Tschoeke, Markus Hellmuth, Arwed Hostmann, Wolfgang Ertel, Andreas Oberholzer

**Affiliations:** 1 Department of Trauma and Reconstructive Surgery, Charité – University Medical School, Campus Benjamin Franklin, Hindenburgdamm 30, 12200 Berlin, Germany

## Abstract

**Background:**

Vertebral fractures resulting from high energy trauma often comprise the risk of posttraumatic degenerative changes in the affected intervertebral discs (IVD). Particularly in conservatively treated patients, or in cases after implant removal of an exclusively posterior stabilization, consecutive disc degeneration and the associated functional losing of the spinal segment clearly represent detrimental treatment results. In this regard, apoptosis of IVD cells has been suggested to be  involved in the critical changes of the extracellular matrix.

**Methods:**

To investigate whether fractures of the vertebrae induce  apoptosis in the affected IVD, disc tissue from patients (n = 17) undergoing  open reduction and internal fixation of thoracolumbar spine fractures were  analysed in regards to caspase activity, apoptosis-receptor expression levels and gene expression of apoptosis-regulating proteins such as Bax and Bcl-2. Healthy IVD tissue (n = 3) obtained from patients undergoing surgical resection of adjacent vertebrae were used as control samples.

**Results:**

In contrast to healthy control IVD tissues, samples from traumatic thoracolumbar IVD showed positive TUNEL staining and a significant increase of caspase-3/7 activity. Interestingly, analyses of the initiator caspase-8 and -9 revealed significantly increased activation levels compared to control values, suggesting the coexistent activation of both the extrinsic (receptor-mediated) and intrinsic (mitochondria-mediated) apoptosis pathway. Accordingly, expression levels of the Fas receptor (FasR) mRNA were significantly increased. Although the TNF receptor I (TNFR I) was only slightly upregulated, corresponding TNFα from trauma IVD presented significantly increased mRNA expression values. Furthermore, traumatic IVD cells demonstrated significantly reduced expression of the mitochondria-bound anti-apoptotic Bcl-2, thereby maintaining baseline transcriptional levels of the pro-apoptotic Bax protein when compared to control IVD cells.

**Conclusion:**

Our data suggest that thoracolumbar fractures induce early caspase-dependent apoptosis in IVD cells of the affected intervertebral disc, in part, by downregulation of the anti-apoptotic protein Bcl-2 (intrinsic apoptosis pathway), as well as signalling via the death receptor complex (TNFR I and FasR).

## Background

Traumatic fractures of the vertebral body, resulting from various forms of severe kinematical impact, often involve local damaging of the associated intervertebral discs (IVD). In addition, previous animal studies and small sample cadaver analyses have demonstrated that abnormal biomechanical loading of the intervertebral segments induced post-traumatic intervertebral disc degeneration [1]. As a consequence, the apparent tissue remodelling in degenerated discs coincides with significant cell matrix changes, including the decrease of collagen content, alteration of collagen distribution, the increase of collagen cross-links and a decrease of proteoglycan levels, which may significantly affect the biomechanical properties of the spinal column. Evidence from non-traumatic degenerated discs further suggest concomitant complex molecular and biological changes [2]. IVD cells play an important role in maintaining disc integrity by producing proteoglycans, type II collagen, and other essential factors involved in the maintenance and repair of the extracellular matrix (ECM), including metalloproteinases, prostaglandins, and nitric oxide [3]. In various states of increased hydrostatic pressure the homeostasis of the ECM metabolism appears to be outbalanced [4,5]. In regards to cell viability in degenerative IVD, previous studies have investigated the importance of apoptosis and a variety of apoptosis-related signalling molecules, such as cytokines and diverse growth factors, which subsequently lead to the collapse or insufficient rearrangement of a functional ECM equilibrium [6,7]. In all mammalian tissues, apoptosis plays an important role in the normal development as well as in tissue and cellular turnover. Apoptosis proceeds, in part, via auto-activation of cytosolic and/or mitochondrial caspases (cystein-containing aspartate-specific proteases), which cleave diverse proteins subsequently resulting in cell auto-destruction. The activation of caspase-3/7 is commonly mediated via the extrinsic pathway by binding to members of the tumour necrosis factor (TNF)-receptor superfamily (e.g. Fas receptor, tumour necrosis factor (TNF) receptor I). Alternatively, the so-called intrinsic mitochondrial pathway  is, in part, influenced by members of the Bcl-family bound to the  mitochondrial membrane, including Bax and Bcl-2, thereby acting as either pro- (Bax) or anti-apoptotic (Bcl-2) regulatory proteins. Previous studies have demonstrated that inhibition of Bcl-2 increases mitochondrial permeability, thus leading to the emission of intramitochondrial proteins into the cytosol (e.g. cytochrome c, endonuclease G (EndoG)), subsequently activating downstream apoptosis-inducing signalling events. More recently, Rannou and colleagues demonstrated that the IVD  degeneration induced by mechanical overload was, in part, mediated via the mitochondrial apoptotic pathway [3]. Furthermore, studies from scoliotic and herniated discs have suggested, that the activation of the Fas/FasL system may play a pivotal role in the progression of IVD degeneration [8,9].

The following study was conducted to investigate whether  thoracolumbar traumatic spine fractures induce apoptosis in the involved  IVD, and if so, which cell-death promoting signalling mechanisms may be  evoked.  

## Methods

### Human intervertebral disc tissue

Fresh IVD specimens were obtained from 17 patients (18 IVD specimens) (mean age 40.6 ± 13.2 years) undergoing open reduction and internal fixation after thoracolumbar spine injury. Healthy IVD from two patients with a primary spinal tumour and one patient undergoing disc prosthesis surgery (mean age 46.0 ± 1.7  years) were obtained in similar fashion and used as control specimens (Table 1 and 2). Tumour infiltration of the respective control IVD specimen was excluded by MR imaging (MRI) and histopathology prior to study enrolment. Degeneration of control IVD was further assessed by T2-weighted MRI signal intensity and graded according to the classification described by Pfirrmann and colleagues [10]. Accordingly, two controls presented with no degeneration (grade I), and one control sample with very low degeneration (grade II), respectively (data not shown). Human disc tissue were immediately stored in liquid nitrogen or RNA Later (Qiagen, Hilden, Germany) after surgical resection. All specimens were collected under informed consent according to our institutional guidelines, and approved by the institutional ethic committee of the Charité – University Medical Schools Berlin (# 226-21).

### RNA isolation and real time RT-PCR analysis

Disc specimens, stored in RNALater at -70°C, were homogenized in Quiazol (Qiagen, Hilden, Germany), and total RNA isolated according to the instruction manual of the Lipid-Tissue-Kit (Qiagen, Hilden, Germany). Quantity and quality of the RNA was evaluated with the RNA 6000 Nano Assay from Agilent Technologies (Agilent Technologies, Waldbronn, Germany). Of the total RNA, 500 ng was denatured at 70°C for 10 minutes in the presence of oligo-primers (pd(T)12–18) (Amersham, Germany) and reverse transcribed into complementary DNA (cDNA) using a 20 μL volume of MMLV-RT (Invitrogen, Germany) complemented with FSB-Buffer (Invitrogen, Germany), 0.5 mmol/L deoxyribonucleotide triphosphates (dNTPs), 10 mmol/L dithiothreitol (DTT), and RNasin (Promega, Mannheim, Germany) at 25°C for 10 minutes, followed by 42°C for 50 minutes. Then, 1-μL aliquots of the resulting cDNA were amplified by RT-PCR in a 25-μL reaction mixture containing the Quantitec Probe RT-PCR Kit (Qiagen, Hilden, Germany) using the TAMRA-labeled method. Validated assays of primer pairs for *TNFR I, TNF, FasR, FasL, Bax *and *Bcl-2 *detection were purchased from Quantitect Primers (Qiagen, Hilden, Germany) and applied according to the manufacturer's instructions. The primer pair of β-Actin housekeeping gene was used as reference control (Quantitect Primers (Qiagen, Hilden, Germany)) and all data normalized to β-Actin gene expression, respectively. The assay was performed in an Opticon I – Real-Time-Cycler from MJ-Research (BioRad, Munich, Germany). The conditions for amplification were as follows: 5 minutes at 94°C, 45 cycles of 30 seconds at 94°C, 30 seconds at 56°C, and 30 seconds at 72°C (as recommended in the manufactures protocol). Results were evaluated as described earlier (Real-Time TaqMan™ PCR Technology 1996, Applied Biosystems GmbH, Weiterstadt, Germany).

### DNA nick end labeling of tissue sections

Tissues stored in liquid nitrogen were cut into cryo-sections of approximately 6 μm thickness and transferred onto Super-Frosted-Slides (Menzel-Gläser, Braunschweig, Germany). Terminal deoxynucleotidyl transferase-mediated deoxyuridine triphosphate-flurescein nick end labelling (TUNEL) was used to detect fragmented DNA known to be associated with apoptotic cell death. End labelling was performed using the DeadEnd Fluorometric Tunel System (Promega, Mannheim, Germany). Counterstaining was performed using Vectashield with DAPI (Alexis, Grünberg, Germany). All stained sections were viewed by epifluorescence microscopy with an Axioskope 40 (Zeiss, Oberkochen, Germany). In general, all assays were performed according to the manufacturer's instructions with minor modifications to optimize staining. Positive controls represented DNase I treated tissue specimens. Negative controls included tissue specimens, which had not been treated with the terminal transferase.

### Caspase-3/7, -8 and -9 activity assay

Apoptotic cell death executioner caspase-3/7 activity, as well as the two classical proximate apoptosis initiator caspases-8 and -9, were determined in tissue homogenates from intervertebral discs. Tissue specimens were homogenized in 25 mM HEPES (pH 7.5), 0.1% NP-40, 5 mM MgCl2, 2 mM DTT, 1.3 mM EDTA, 1 mM EGTA and CompleteMini (Roche, Penzberg, Germany). The homogenates were centrifuged at 50.000 *g*, and all supernatants removed for the assay of caspase-3/7, -8 or -9 activity, respectively, by using the Apo-ONE Homogenous caspase-3/7 assay or the Caspase Glow (caspase-8, -9) assay (Promega, Mannheim, Germany). All preparations were applied according to the manufacturer's instructions. The intensity of the emitted fluorescence was determined after 16 h incubation with a GeniusSpectra Fluorplus fluorescence spectrometer (Tecan Instruments, Crailsheim, Germany), at an excitation wavelength of 485 nm, and an emission wavelength of 535 nm.

### Data presentation and statistical analysis

All figure data is presented as the mean value and the standard error of the mean (Data ± SEM). Real-time RT-PCR data is presented in log scale and shows relative expression levels (REL) as normalized to β-Actin gene expression. Statistical significance for all comparison analysis was assumed at *P *< 0.05 using the Student's *t *test.

## Results

### Spinal trauma induces apoptosis in thoracolumbar intervertebral discs (IVD)

**Figure 1:**
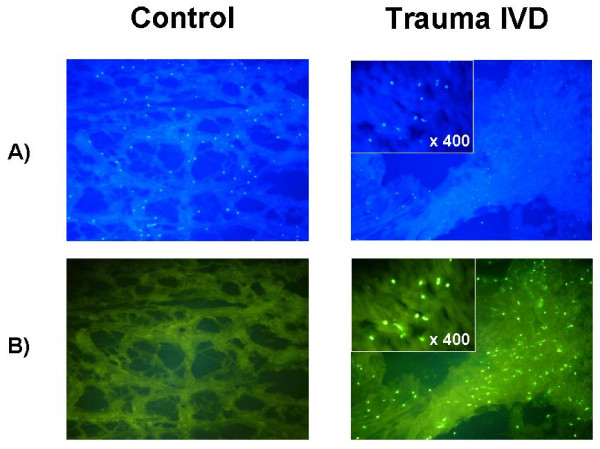
**Trauma-induced IVD apoptosis**. Large images displayed at × 100 magnification. A) DAPI staining positive for IVD cells in the disc tissue matrix (control (left panel) versus trauma (right panel)). B) TUNEL staining demonstrates positive DNA fragmentation of IVD cells in the trauma IVD tissue sample (right panel) versus the negative control tissue (left panel).

**Figure 2:**
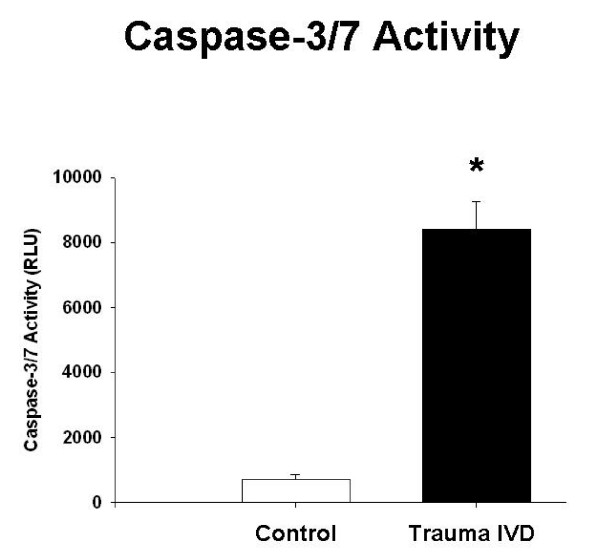
**Caspase-3/7 activity is increased in trauma IVD cells**. Analysis of the executioner caspase-3/7 activity demonstrates a significant increase in trauma IVD cells compared to levels determined in control IVD samples. * p < 0.001 as determined by Student's t test (trauma vs. control).

Since IVD cell matrix degeneration predominantly occurs in cells producing collagen type II, all specimens were verified to be collagen type II positive by immunhistological staining analyses prior to further analytical processing (data not shown). However, the region of IVD cell origin (nucleus pulposus versus annulus fibrosus) was not further specified due to the trauma induced gross morphological damage. To assess the overall rate of apoptosis in respective IVD cells a fluorometric TUNEL staining analysis was performed in both trauma and control IVD tissue. Interestingly, traumatic thoracolumbar IVD showed an explicit increase of TUNEL positive cells, indicating evident apoptosis in comparison to negative control samples (Figure 1B). This observation was subsequently confirmed by analyses of the apoptosis executioner caspase-3/7 activity. Thus, IVD cells from traumatic disc specimens demonstrated a significant increase of caspase-3/7 activity (RLU 8404.3 ± 2463.3) when compared to healthy control samples (RLU 706.1 ± 287; p < 0.001; Figure 2).

### Caspase -8 and -9 activity is increased in traumatic IVD cells

**Figure 3:**
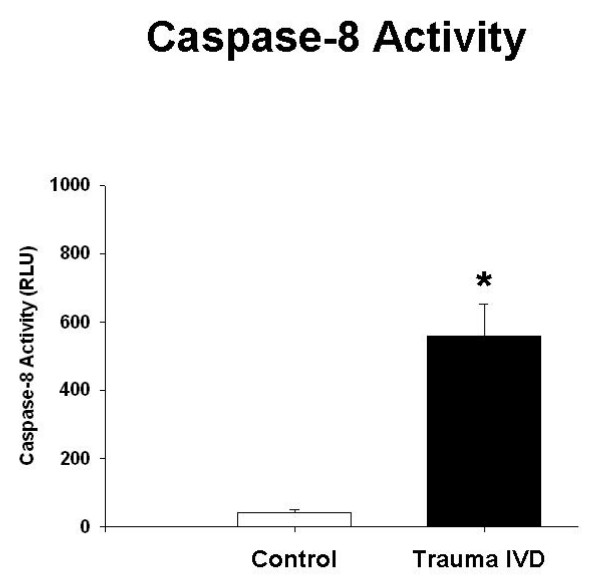
**Trauma-induced increase of caspase-8 activity in IVD cells**. Analysis of the initiating caspase-8 within the extrinsic receptor-mediated apoptosis pathway revealed a significant increase compared to non-traumatic control IVD. * p < 0.05 as determined by Student's t test (trauma vs. control).

In evaluation of the apoptosis initiating event, we determined the activity of the two classical initiator proteases, namely caspase-8 (extrinsic receptor-mediated apoptosis pathway) and caspase-9 (intrinsic mitochondria-mediated apoptosis pathway). Interestingly, spinal trauma apparently increased both the extrinsic caspase-8 activity (RLU 558.4 ± 93.9) as well as the intrinsic caspase-9 activity (RLU 1975.2 ± 394.7) in respective IVD cell samples from fractured thoracolumbar segments when compared to non-traumatic control IVD (control caspase-8: RLU 40 ± 11.5; control caspase-9: 164 ± 75.7; p < 0.05; Figures 3+4).

### Anti-apoptotic Bcl-2 protein is downregulated in traumatic thoracolumbar intervertebral discs (IVD)

**Figure 5:**
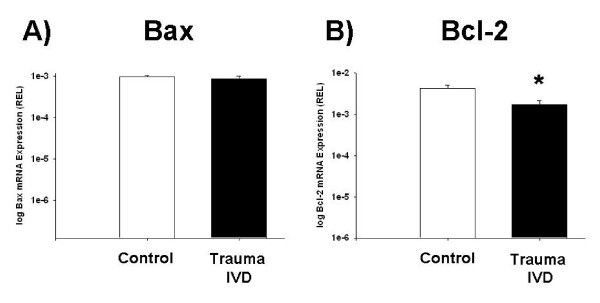
**mRNA expression levels of the pro-apoptotic Bax and anti-apoptotic Bcl-2 protein**. A) Expressions of Bax mRNA in traumatic IVD cells did not differ from those determined in non-trauma control samples. B) Bcl-2 mRNA reveals a significant downregulation in traumatic IVD compared to control IVD cells. * p < 0.05 as determined by Student's t test (trauma vs. control).

Based on our observation of increased apoptosis and the activation of caspase-9 in traumatic IVD cells, we evaluated the gene expression of two key apoptosis-regulating proteins known to stabilize the mitochondria within the intrinsic apoptosis pathway such as the pro-apoptotic Bax and anti-apoptotic Bcl-2. Gene expression of the pro-apoptotic Bax did not differ from healthy control samples (Figure 5A). In contrast, traumatic thoracolumbar IVD cells demonstrated a significant decrease in gene expression of anti-apoptotic Bcl-2 compared to normal control specimens (p < 0.05; Figure 5B).

### Both TNFRI/TNFα and FasR/FasL mediated signalling are involved in trauma-induced intervertebral disc (IVD) apoptosis

**Figure 6:**
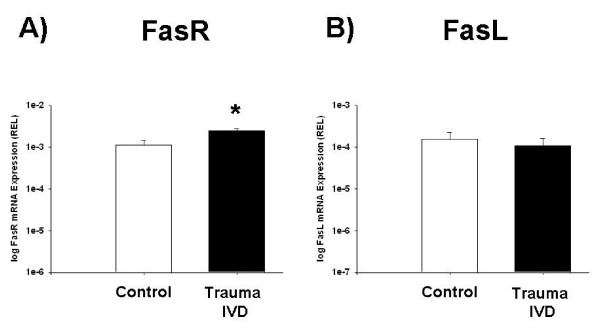
**mRNA expression levels of the Fas receptor (FasR) and Fas ligand (FasL) in traumatic versus control IVD cells**. A) FasR mRNA expression levels in traumatic IVD cells significantly differed from non-traumatic control IVD cells. B) Analysis of the FasL mRNA expression in traumatic IVD cells revealed expression levels comparable to non-traumatic control IVD cells. * p < 0.05 as determined by Student's t test (trauma vs. control).

**Figure 7:**
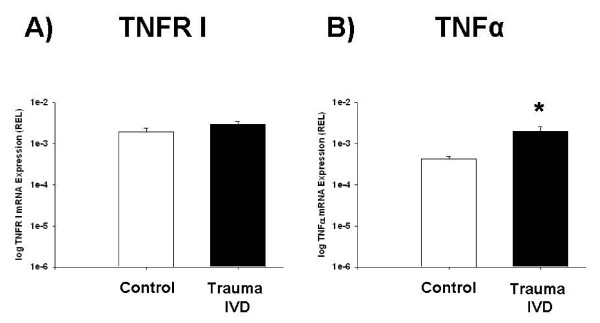
**mRNA expression levels of the TNF receptor I (TNFR I) and TNFα in traumatic versus control IVD cells**. A) TNFR I mRNA expression levels in traumatic IVD cells demonstrated increased values compared to control IVD cells. B) Analysis of the TNFα mRNA expression revealed significantly increased expression levels in traumatic IVD cells when compared to non-traumatic control IVD cells. * p < 0.05 as determined by Student's t test (trauma vs. control).

In accordance with the increased apoptosis initiator caspase-8 activity determined in traumatic IVD cells, the analyses of the cell death receptor FasR mRNA revealed significantly increased expression values when compared to non-traumatic controls (p < 0.05) (Figure 6A). However, the mRNA expression of the corresponding Fas ligand in trauma patient IVD cells demonstrated values rather equal to the expression levels determined in control samples (Figure 6B). Along the lines of receptor-mediated signalling of apoptosis, analysis of the TNFR I mRNA expression also revealed a modest upregulation when compared to normal control samples (Figure 7A). Moreover, mRNA expression of TNFα was significantly upregulated in traumatic IVD (p < 0.05).

## Discussion

Spinal trauma, associated with vertebral fractures, seems to mark an important trigger mechanism for both morphological and molecular changes of the respective IVD matrix. Findings from follow-up studies investigating the loss of reduction and functional outcome in conservatively treated patients, and patients after implant removal of an exclusively posterior stabilized fracture segment, demonstrated significant kyphosis and functional losing, when compared to age-matched controls or earlier post-operative results, respectively [11-13]. Although there is increasing evidence that apoptosis appears to play a central role in IVD tissue degeneration, most studies have demonstrated this process to be rather coherent with chronic and prolonged abnormal mechanical loading, including scoliotic deformities [9,14]. Previous studies investigating IVD chondrocytes and fibrocytes in mice demonstrated increased apoptosis in load challenged cells, suggesting a strong correlation of the load amplitude and duration with the degree of disc degeneration [15-17]. In consideration of these findings it may be hypothesized, that acute trauma to the thoracolumbar spine can induce a similar reaction in IVD, in addition to a potentially gross morphological tissue damage associated with the fractured vertebral body. Therefore, our study intended to investigate apoptosis as a potential mechanism underlying post-traumatic IVD degeneration. Hence, we demonstrate here that trauma induces apoptosis in thoracolumbar IVD cells from fractured spinal segments, as confirmed by both a positive TUNEL staining and the significant increase of the executioner caspase-3/7 activity in comparison to non-apoptotic control IVD samples. Furthermore, our mRNA analysis of the traumatic IVD cells demonstrated a significant downregulation of the anti-apoptotic Bcl-2, which is consistent with previous findings from Rannou and colleagues describing the mitochondrial apoptotic pathway to be involved in IVD degeneration after mechanical-stress [3]. In this regard, our data strongly suggest that downregulation of key apoptosis-regulating proteins, such as Bcl-2, promotes mitochondrial membrane permeability, which subsequently leads to activation of the intrinsic caspase-dependent pathway via caspase-9 and, finally, caspase-3/7 mediated apoptosis (Figure 2+4) (for review see [18] and [19]). Furthermore, studies investigating the regulation of cartilage matrix genes in chondrocytes have suggested an important coordinating role of Bcl-2, by demonstrating that down-regulation of collagen type II was closely related to a substantial reduction of Bcl-2 protein levels [20]. Furthermore, our data endorse the supposition that apoptosis in traumatic IVD cells may be augmented via the extrinsic TNFα and TNFR I mediated pathway by activating the initiator caspase-8, and subsequently caspase-3/7 (Figure 2+3). A recent study by Séguin and colleagues reported TNFα, even at low physiologically present concentrations, to increase gene expression of matrix metalloproteinases (MMPs) and ADAM-TS (a disintegrin and metalloproteinase with thrombospondin motifs) known to deplete tissue proteoglycans in the early stages of IVD degeneration [21]. In contrast, studies from scoliotic and herniated discs have suggested the activation of the Fas/FasL system to play a pivotal role in the progression of apoptosis-associated IVD degeneration [8,9]. In accordance with reports from degenerative and scoliotic IVD [22,23], our results further complement these data by demonstrating distinctively upregulated mRNA expressions of the FasR in IVD from trauma patients compared to healthy control specimens. Thus, it is likely that not only consistent and prolonged abnormal mechanical loading, but also the acute trauma may initiate a receptor-mediated cell death mechanism subsequently leading to premature IVD degeneration. Although it must be acknowledged that these observations solely reflect the transcriptional level, and thus represent a critical limitation to our study, these regulatory changes argue strongly in favour of an active IVD cell response to a traumatic stimulus in associated IVD tissue. In addition, recent publications attempting to illuminate this molecular aspect in the pathophysiology of human IVD degeneration, including scoliosis and disc herniation or prolaps, have exclusively lacked control samples and alternative analyses on a protein level, mainly due to the nature and availability of tissue collection and quality [24,25]. Although the exact signalling mechanisms of these  interactions in IVD cells remain to be elucidated in further detail, our  findings support the common notion that pro-inflammatory mediators have an important impact on IVD cell matrix changes, including the potential to promote post-traumatic degeneration [26].

## Conclusion

Taken together, our findings strongly suggest that apoptosis contributes to the reduction of matrix integrity in traumatic IVD via a caspase-dependent mechanism. Furthermore, this study demonstrates for the first time in humans, that the upregulation of both cell death receptors TNFR I and FasR, as well as the downregulation of anti-apoptotic regulator proteins such as Bcl-2, may postulate for post-traumatic changes in intracellular matrix integrity.

## Competing interests

The author(s) declare that they have no competing interests.

## Authors' contributions

Christoph-E. Heyde supervised the study, analysed the data, carried out the statistical analysis and wrote the manuscript.

Sven K. Tschoeke enrolled patients, collected samples, analysed the data, carried out the statistical analysis and wrote the manuscript.

Markus Hellmuth prepared the samples, analysed the data and provided advice in interpreting all analysis.

Arwed Hostmann provided advice and interpretation of the data analysis.

Wolfgang Ertel provided intellectual input and critically reviewed the manuscript.

Andreas Oberholzer provided intellectual input, advice in interpreting the analysis and critically reviewed the manuscript.

## Pre-publication history

The pre-publication history for this paper can be accessed here:



## Figures and Tables

**Figure 4:**
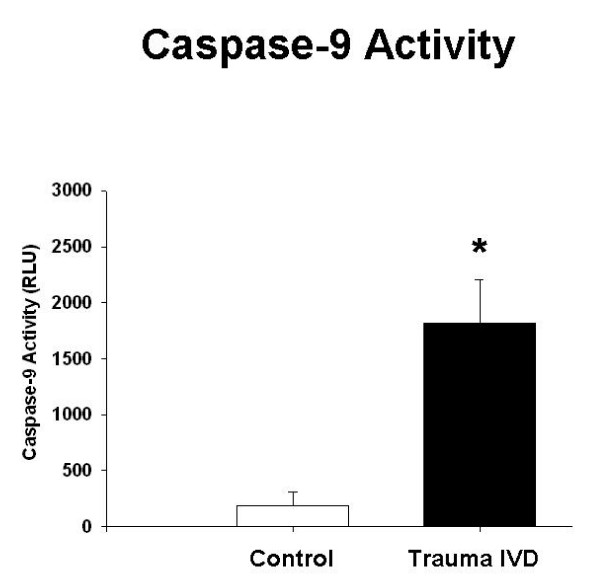
**Trauma-induced increase of caspase-9 activity in IVD cells**. Analysis of the initiating caspase-9 within the intrinsic mitochondria-mediated apoptosis pathway revealed a significant increase compared to non-traumatic control IVD. * p < 0.05 as determined by Student's t test (trauma vs. control).

**Table 1:** Patient Demographics (data presented as mean ± SD).

	**Control Patients**	**Trauma Patients**
**Subjects (n)**	3	17
**Male/Female (n)**	2/1	12/5
**Age (years)**	46.0 ± 1.7	40.6 ± 13.2

**Table 2:** Clinical Data.

	**Age**	**Sex**	**Diagnosis**	**Vertebra**	**Fracture Type**	**IVD Segment**
**Control**	47	female	Hemangioma L3	L 3	/	L 2/3
	44	male	Degeneration L4/5 *	L 4	/	L 3/4
	47	male	Sacral Chordoma	S 1	/	L 5/S 1
**Trauma**	27	male	Fracture	TH 5	A 3.1	TH 4/5
	21	male	Fracture	TH 6	C 1	TH 4/5
	54	female	Fracture	TH 5	B 1.1	TH 5/6
	39	male	Fracture	TH 8	B 1.2	TH 7/8
	28	male	Fracture	TH 10	A 3.1	TH 9/10
	39	male	Fracture	TH 12	A 3.1	TH 11/12
	31	male	Fracture	L 1	A 3.1	TH 12/L 1
	43	male	Fracture	L 2	B 2.2	TH 12/L 1
	28	male	Fracture	L 1	A 3.1	TH 12/L 1
	59	male	Fracture	L 1	A 3.1	TH 12/L 1
	48	female	Fracture	TH 12	B 1.1	TH 12/L 1
	44	female	Fracture	L 1	A 3.1	TH 12/L 1
	59	female	Fracture	L 1	A 3.2	TH 12/L 1
	26	female	Fracture	L 1	A 3.2	L 1/2
	47	male	Fracture	L 3	A 3.3	L 2/3
			Disc Disruption	TH 12/L 1	B 1.1	TH 12/L 1
	64	male	Fracture	L 3	A 3.2	L 2/3

* Adjacent proximal disc resected after intraoperative fracture of the 4th lumbar vertebra within the attempt of disc prosthesis implantation in the L 4/5 segment.
